# Balanced Ambipolar OECTs through Tunability of Blend Microstructure

**DOI:** 10.1021/acsami.5c05400

**Published:** 2025-07-18

**Authors:** Noam Moscovich, Sasha Simotko, Efrat Reyn, Ido Zerachia, Amit Hadar, Gitti L. Frey

**Affiliations:** † Department of Materials Science and Engineering, 26747 Technion − Israel Institute of Technology, Haifa 32000, Israel; ‡ The Nancy and Stephen Grand Technion Energy Program, Technion − Israel Institute of Technology, Haifa 32000, Israel

**Keywords:** organic mixed ionic electronic conductors, organic electrochemical transistor, blends, ambipolarity, microstructure

## Abstract

Organic electrochemical transistors (OECTs) are promising building blocks for bioelectronics, bridging ionic biological signals and electronic circuits. Ambipolar OECTs, capable of both n- and p-type charge transport, are highly desirable for versatile bioelectronic applications, offering a simplified circuit design and enhanced sensing capabilities. However, achieving a balanced ambipolar performance remains a materials science challenge. Here we show that blending judiciously selected unipolar p-type and n-type organic mixed ionic-electronic conductors (OMIECs), and most importantly, tuning film microstructure through composition and thermal treatment, can be leveraged to attain balanced ambipolar OECTs with near-equal n-type and p-type performances, both in the transconductance and in symmetrical threshold voltages. This is demonstrated by studying two blends based on a p-type OMIEC polymer and two n-type OMIEC fullerene derivatives with distinct miscibility and self-assembly tendencies that direct completely different blend organizations. Employing comprehensive electrochemical and microstructural characterization, we were able to correlate microstructure features, such as phase separation, domain continuity, and crystallinity, with volumetric capacitance and charge mobility, and hence overall device performance in both polarities. Based on the insights gained in this study, we propose a set of design rules and a rational framework for realizing balanced ambipolar OECTs using the blend approach. These rules emphasize informed material selection and precise microstructure control of phase continuity and order, attainable through composition and thermal annealing. The blend strategy offers a facile and versatile pathway for advancing next-generation bioelectronic devices with multifunctional OECT performance through rational microstructure engineering.

## Graphical abstract

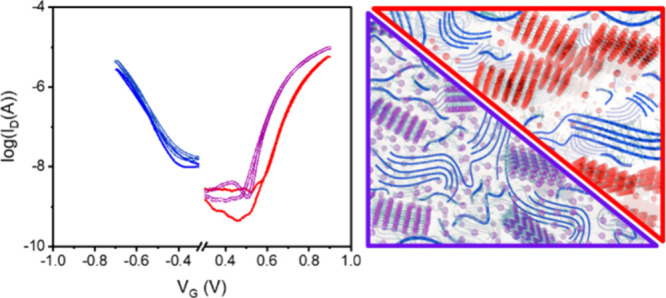

## 1 Introduction

The integration of organic electronic devices with biological systems has revolutionized the field of bioelectronics by harnessing the unique properties of organic semiconductors, such as mechanical flexibility, softness, and biocompatibility, to create devices that seamlessly interface with tissues, living cells, and biofluids.^1–3^ Particularly, organic electrochemical transistors (OECTs) have emerged as key bioelectronic devices capable of transducing ionic signals, which are ubiquitous in biological systems, into electronic signals available for logic circuits and other computational systems. OECTs offer high sensitivity and low-voltage operation, making them particularly well-suited for biosensing, neural interfacing, and additional bioelectronic applications where real-time, low-power signal transduction is crucial.^4–10^

The coupling between ionic and electronic signals in OECTs is enabled by the utilization of Organic Mixed Ionic-Electronic Conductors (OMIECs), i.e., materials that support both ionic and electronic charge transport and couple between these charge transport processes, as the active channel material of the transistor.^11^ The chemical structure of the OMIECs is generally that of known organic semiconductors with extended conjugation to support charge transport and solubilizing side chains that also support ion transport. The presence of conjugated donor groups enhances the transport of holes for p-type performance, and acceptor groups lead to n-type performance. Ambipolarity, i.e., the ability to conduct both electrons and holes in a single material, may be achieved by combining donor and acceptor moieties. However, the synthesis is laborious with one polarity generally suppressing the other.^12,13^

Ambipolar OECTs, however, have become a focal point of active research, reflecting a growing interest in their potential^14–18^ for simplifying circuit fabrication and offering sensing of both positive and negative ions in a single device.^19,20^ Fully balanced ambipolarity, expressed in similar output currents for both polarities and symmetrical threshold voltages vs the undoped state of the device, is desirable for reconfigurable complex logic circuit design and operation, and allows for low power consumption. Ambipolar OMIECs enable ambipolar OECT performance, but the overall performance is limited by the imbalance between the polarities, with one polarity generally coming at the expense of the other.^21,22^ Recently, a new strategy for ambipolar OECTs based on blending p-type and n-type unipolar OMIECs was suggested by Leong and co-workers^23,24^ and us.^25,26^ The blend approach capitalizes on a broad collection of available materials with no need for further complex synthesis and the ability to direct the performance through material selection and blend composition.

The blend approach is widely used in organic electronics, effectively unlocking functionalities that are inaccessible with a single material, thereby expanding device capabilities while maintaining simple and standard fabrication. For example, organic solar cells (OSCs) are composed of donor:acceptor blends for efficient charge generation, separation, and transport. It is well established that the microstructure of the blend, including domain purity, size, distribution, and continuity, in addition to the degree of crystallinity and order, significantly influences charge generation, separation, and transport efficiencies, ultimately determining the overall efficiency of the solar cells.^27–29^ Extensive and rigorous investigation of the polymer:fullerene system revealed the optimal bulk heterojunction (BHJ) morphology for OSCs, and how blend composition and processing conditions can be manipulated to direct the desired microstructure and enhance the overall performance.^30–35^

In the study of OECTs, while significant advancements have been achieved, the understanding of the relationship between microstructure and performance is still evolving. It was revealed that microstructural features, such as the distribution of amorphous and crystalline domains and the degree of crystallinity and orientation, are of paramount importance in dictating the key processes in OECTs, including ionic infiltration and expulsion, electronic charge transport, and ionic-electronic coupling.^36–38^ Moreover, morphological features, such as domain connectivity and spatial organization, have been correlated with critical device metrics, including transconductance and response time.^36,38–40^ These findings manifest the importance of establishing the relationship between the microstructure and performance in the OECTs, hence providing a framework for further exploration and optimization of these systems by means of microstructural engineering.

Utilizing the blend approach in OECTs can yield valuable insights into the relationship between microstructure and performance, while also enabling functionalities that cannot be achieved with a single-material device.^23,25^ Our recent work demonstrated that blending a p-type OMIEC polymer with an n-type OMIEC fullerene results in ambipolar OECT performance. However, the composition of the blend in that study was predominantly n-type fullerene, which limited the ability to systematically investigate the impact of composition on the microstructure and performance. In this study, we perform a rigorous analysis of blend composition-microstructure-performance and show that microstructure tunability through composition and thermal treatment can lead to fully balanced ambipolar OECT performance. To demonstrate this, we selected two n-type fullerene OMIEC derivatives and studied their blends with a benchmark p-type OMIEC polythiophene. The distinct tendency of each fullerene concerning phase separation and crystallization leads to completely different microstructure evolution as a function of composition and thermal treatments. By combining detailed electrochemical analysis with microstructural characterization, we highlight the critical influence of microstructure on the performance of the OECT, identifying limiting factors and synergistic effects and achieving fully balanced ambipolar performance. Finally, we draw design rules to create a framework for the rational selection of OMIEC blends for general microstructure control and specifically ambipolar performance.

## 2 Experimental/Methods

### 2.1 Materials

Poly­(3-[2-(2-methoxyethoxy) ethoxy]­ethylthiophene-2,5-diyl) regioregular (P3MEEET, Mn = 8 kDa Mw = 12 kDa, Pd = 1.6, RR = 87%, Rieke Metals, Lincoln, NE), 2′-[4″-((((2-ethoxy)-2-ethoxy)-2-ethoxy)-2-ethoxy)­phenyl]-fulleropyrrolidine (PTEG-1, Solenne BV), 2′-[2″,3″,4″-tris­(((2-methoxy)-2-ethoxy)-2-ethoxy)­phenyl]-fulleropyrrolidine (PrC60MA, Lumtec), KCl (Sigma-Aldrich Israel Ltd.), Diethylzinc (Sigma-Aldrich Israel Ltd.), and anhydrous chloroform (which includes amylene for stabilization, ≥99%, Sigma-Aldrich Israel Ltd.) were purchased and used as received.

### 2.2 Film Preparation

Substrates were cleaned in acetone, methanol, and isopropanol for 15 min each in an ultrasonic bath, followed by drying under nitrogen (99.995%). All solution preparation and film deposition processes were performed under an inert nitrogen atmosphere. P3MEEET, PTEG-1, and PrC60MA were dissolved in anhydrous chloroform at concentrations of either 10 or 30 mg/mL at ambient temperature. Blend solutions were prepared by mixing the component solutions at wt/wt % ratios of 25:75, 50:50, and 75:25 to achieve a 10 mg/mL total concentration. Films were spun at 1000 rpm for 60 s, followed by 3000 rpm for 10 s at RT. Thermally annealed (TA) films were annealed on a hot plate at 120 °C for 20 min. Film thicknesses were measured using a Bruker DektakXT stylus profilometer with a needle tip radius of 12.5 μm and were found to be ∼75 nm.

### 2.3 Electrochemistry

For cyclic voltammetry (CV) and spectroelectrochemistry (SEC), films were spun on FTO-coated glass substrates (surface resistivity of ∼7 Ω/sq, Sigma-Aldrich Israel Ltd.) and used as the working electrodes (WE) in a 0.1 M KCl solution. A Pt wire served as the counter electrode (CE), and a Ag/AgCl pellet (E206 Warner Instruments, LLC) served as the reference electrode (RE). SEC was measured using a UV–vis spectrophotometer (Cary 100 Scan, Agilent Technologies, Inc.), with the electrodes connected to an external potentiostat (Palmsens4) applying 0.05 V potential steps from 0.7 to −0.9 V and held for 100 s each during spectrum acquisition. Absorbance changes were calculated with respect to the 0 V spectrum. In CV measurements, a potential sweep of 0.0 V → 0.7 V → −0.9 V → 0.0 V (V_WE_) was repeated for 20 cycles at a scan rate of 0.1 V/s, with voltage steps of 1 mV. All CV and SEC measurements were performed under ambient conditions.

For electrochemical impedance spectroscopy (EIS) measurements, gold-coated glass substrates with a gold WE area of 0.0036 cm^2^ were coated as previously described and immersed in a 0.1 M KCl solution with Pt and Ag/AgCl as the CE and RE, respectively. Au was used so that the C* values can be directly compared to the transconductance obtained from the OECTs using similar Au contacts, and all measurements were performed under ambient conditions. Measurements were recorded using a Palmsens4 potentiostat at a set of direct current (DC) potential biases (between −0.9 and 0.7 V vs Ag/AgCl) and an alternating current (AC) perturbation wave with an amplitude of 10 mV and a frequency range of 0.1–10^6^ Hz. Analysis was performed based on an average across at least three devices for each material composition. The volumetric capacitance was extracted at a frequency of 1 Hz and at the maximum doping potential for both polarities (−0.9 V for n-type and 0.7 V for p-type vs Ag/AgCl) using $C^{*}=\frac{1}{2\pi^{*}f^{*}\left|Z^{img}\right|}$ , where *f* is the frequency, and *Z* ^img^is the imaginary part of the impedance.

### 2.4 Device Fabrication and Testing

Au (50 nm)/Cr (5 nm) were thermally deposited under a vacuum of approximately 6 × 10^–6^ Torr (Edwards Auto 500) through an Ossila Ltd. (E291) shadow mask with channel dimensions of 1000 μm W and 30 μm L onto ultraflat quartz-coated glass substrates (cleaned as previously described). The patterned substrates were cleaned, blow-dried, and coated with organic film. The film was dry-wiped from most of the metal contacts, except for the channel area, to minimize device crosstalk. Kapton tape was used to manually mask the areas where the metal contacts were exposed to avoid contact with the electrolyte.

For the OECT device characterization, a dual-channel source measure unit (SMU) (B2902A, Keysight Technologies, Inc.) was used with dedicated software (EasyExpert group+). The SMU was connected to source and drain gold contacts as well as a Ag/AgCl pellet (E206 Warner Instruments, LLC) that was used as the gate electrode. The electrolyte was 0.1 M KCl and was dropped onto the devices before measurement. Output and Transfer curves were acquired with a double-sweep measurement. For output curves, the drain voltage (*V*_D_) was swept in steps of 13 mV at a scan rate of 0.1 V/s, in −0.05 V → 0.6 V for n-type scan and 0.05 V → −0.6 V for p-type scan, at constant gate voltages (*V*_G_) ranging from 0 to 0.9 V for n-type scan and 0 to −0.7 V for p-type scan, with a step size of 0.05 V. For transfer curves, the *V*_G_ was swept in steps of 1 mV at a scan rate of 0.1 V/s, in 0 V → 0.9 V for the n-type scan and 0 V → −0.7 V for the p-type scan, at a constant *V*_D_ of 0.3 V or −0.3 V for n-type and p-type scans, respectively. The measurement sequence for each device was: Output (p-type) → Transfer (p-type) → Output (n-type) → Transfer (n-type), repeated for 3 cycles. Analysis was performed based on an average across at least five devices for each material composition, with each device result averaged over three operational cycles. The threshold voltage (*V*_th_) was determined from the transfer characteristics by applying linear regression to the square root of the drain current (*I*_D_) versus the gate voltage (*V*_G_). The maximum transconductance (*g*_m_), calculated as the derivative of *I*_D_ with respect to *V*_G_, is derived from the transfer characteristics recorded at a set drain voltage of ±0.3 V (depending on the polarity). All of the OECT measurements were performed under ambient conditions.

### 2.5 Microstructure Analysis

For microstructure characterization (VPI, HRSEM, and GIWAXS), organic films were spun on Si substrates. Vapor Phase Infiltration (VPI) of Zinc Oxide was performed using an Atomic Layer Deposition (ALD) system (Ultratech/Cambridge Nanotech Savannah, Veeco Instruments Inc.) at a temperature of 60 °C through 80 alternating pulses of diethylzinc (DEZ) and deionized (DI) water, with nitrogen as carrier and purge gas. Each cycle included two DEZ pulses (0.02 s pulse +20 s hold +25 s purge) and two DI water pulses (0.04 s pulse +20 s hold +25 s purge). The flow rate of the reactor was 20 sccm. After VPI, the substrates were cleaved in liquid nitrogen for cross-section HRSEM imaging using a Zeiss UltraPlus FEG-SEM equipped with a backscattered electrons (BSE) detector at 1.5 kV accelerating voltage and ∼2.7 mm working distance.

GIWAXS measurements of silicon-coated substrates were performed using a Rigaku SmartLab 9 kW X-ray diffractometer with Cu Kα radiation (λ = 1.54186 Å), equipped with a HyPix-3000 2D detector and with an installed aperture slit and reflection attachment head. The measurements were recorded at an incident angle of ω = 0.19°. The out-of-plane line cut was taken at *q*_*xy*_/2π = 0 nm^–1^ and the in-plane line cut at *q*_*z*_/2π = 0.04425 nm^–1^.

## 3 Results

Building on the extensive investigation of polythiophene:fullerene blends for OSCs and the successful implementation of OMIEC polymer:fullerene blends for ambipolar OECTs, we selected blend systems based on an OMIEC polythiophene and an OMIEC fullerene derivative. The chemical structures of the chosen materials are depicted in Figure 1a. P3MEEET (Poly­(3-[2-[2-(2-Methoxyethoxy)­ethoxy]­ethyl]­thiophene-2,5-diyl)) is a well-characterized unipolar p-type polythiophene^41–43^ with the same backbone as the benchmark organic semiconductor P3HT. The ethyl spacer mitigates the influence of the electro-withdrawing effect of the glycolated side chains on the polythiophene backbone, so the HOMO energy level position of P3HT, ∼5.2 eV, is maintained.^44–48^ P3MEEET demonstrated very good unipolar p-type OECT performance with C* value of 242 F/cm^3^, *V*_th_ of −0.57 V, and a *g*_m_ of 20.4 S/cm (μC* of 11.5 F/cmVs).^41^ Notably, it was shown that the average molecular weight of P3MEEET plays a critical role in governing the device performance.^42^

**Figure 1:**
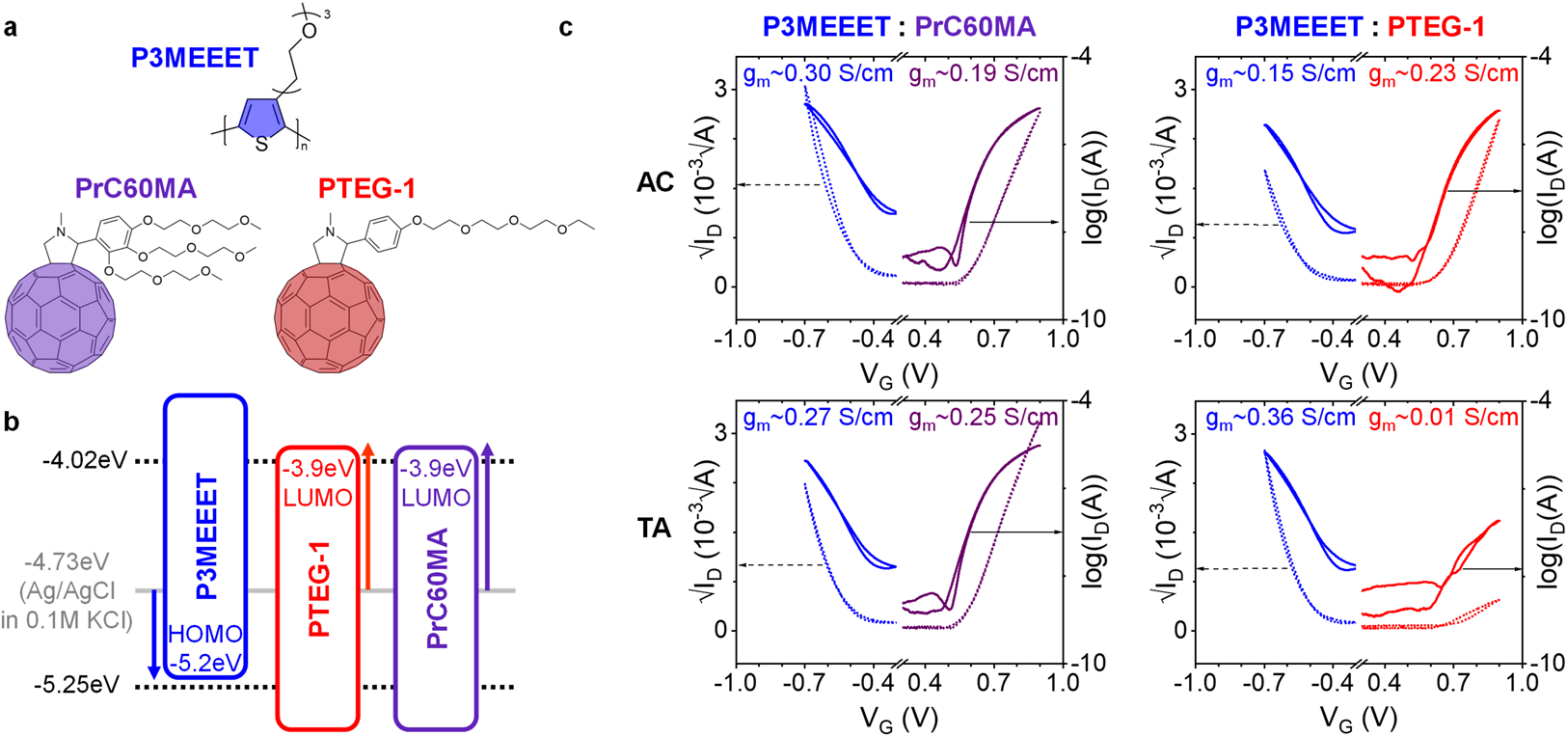
Material selection and 50:50 blend device characteristics: Chemical structures (a), and energy level scheme (b) of the materials used in this study. (b) also shows the approximated energy level of the Ag/AgCl reference electrode and the stable water electrochemical window (all vs vacuum level).^51^ The arrows in (b) represent the working range for the p- and n-type components. (c) Transfer characteristics (n-type sweeps in purple and red, p-type in blue) and the extracted transconductance values (*g*_m_) of the as cast (AC) and thermally annealed (TA) (20 min @ 120 °C) 50:50 blends of the OECT devices.

The two fullerene derivatives, PTEG-1 and PrC60MA (2′-[4″-((((2-ethoxy)-2-ethoxy)-2-ethoxy)-2-ethoxy)­phenyl]-fulleropyrrolidine and C60,*N*,*N*,*N*-trimethyl-1-(2,3,4-tris­(2-(2-methoxyethoxy)­ethoxy)­phenyl)­methanaminium monoadduct, respectively) are OMIEC equivalents of PCBM and, hence, possess similar lowest unoccupied molecular orbital (LUMO) levels of ∼3.9 eV.^49^ Previous studies utilized these OMIEC fullerenes in unipolar n-type OECT devices where PrC60MA exhibited a C* of 61 F/cm^3^, *V*_th_ of 0.62 V, and *g*_m_ of 6.1 S/cm (μC* of 21.7 F/cmVs),^25^ while PTEG-1 showed a C* of 40 F/cm^3^, *V*_th_ of 0.84 V, and *g*_m_ of 4.6 S/cm (μC* of 0.1 F/cmVs).^50^

These materials were selected for ambipolar blends following these design rules: (i) the energy levels, i.e. HOMO of the polymer and LUMO of the fullerene, are within (or slightly beyond) the water electrochemical window, as shown in Figure 1b, ensuring stable electrochemical doping in aqueous environments; (ii) the energy gap between the levels is large, 1.3 eV, effectively preventing charge recombination; (iii) The energy level alignment is symmetric around the Ag/AgCl electrode working conditions potential offering an “OFF” state at 0 V, (iv) The μC* values are of the same order of magnitude suggesting relatively balanced blend compositions, and (v) Fullerene degree of crystallinity is known to direct distinct microstructures^52–54^ so the selection of OMIEC fullerenes with different degrees of crystallization and self-packing can be used to tune the overall microstructures of the blend to allow structure–property correlations. Earlier studies showed that PTEG-1 is highly crystalline upon film deposition, with its glycol side-chains interdigitating.^55^ On the other hand, fullerene derivatives with more or longer glycolated side chains exhibited increased interlayer spacing and less order that improved upon annealing.^56,57^ Indeed, as deposited films of PrC_60_MA show a larger interlayer spacing and lower degree of order, compared to PTEG-1, that significantly improves upon annealing.^25,58^

To test our judicious materials selection and the suggested approach of the OMIEC blends for balanced ambipolar OECTs, we fabricated and analyzed the OECTs based on 50:50 wt % blends of P3MEEET:PrC60MA and P3MEEET:PTEG-1, as cast (AC) and after thermal annealing (TA) (Figure 1c). In contrast to the unipolar behavior of the pristine materials (Figure S1), the 50:50 blend devices, AC and TA, exhibit clear ambipolar OECT performances. Furthermore, the TA P3MEEET:PrC60MA blend and the AC P3MEEET:PTEG-1 blend show fully balanced ambipolarity with nearly identical transconductance *g*_m_ values for both n-type and p-type as well as symmetrical threshold voltages, as shown in Table 1. These results confirm the suitability of the above criteria for materials selection. However, while the AC and TA P3MEEET:PrC60MA devices exhibit similar performances, the performance of the P3MEEET:PTEG-1 blend is significantly reduced upon annealing. Therefore, to specifically identify the effects of blend composition and thermal treatment on the electrochemical processes, it is necessary to examine OECT behavior across all blend ratios before and after the thermal treatment.

**Table 1:** Summary of Fully Balanced Ambipolar Blend-Based OECT Characteristics

blend	polarity	*g*_m_ [S/cm]	*V*_th_ [V]
TA 50:50 P3MEEET:PrC60MA	n	0.25 ± 0.05	+0.53 ± 0.01
	p	0.27 ± 0.07	–0.53 ± 0.01
AC 50:50 P3MEEET:PTEG-1	n	0.23 ± 0.09	+0.67 ± 0.02
	p	0.15 ± 0.04	–0.55 ± 0.01

OECT output and transfer curves for P3MEEET:PrC60MA (Figures S2 and S3) and P3MEEET:PTEG-1 (Figures S4 and S5) before and after the thermal treatments were measured, and the transconductance values, *g*_m_, at both polarities extracted from the transfer curves and summarized in Figure 2. For P3MEEET, PrC60MA, and all their blend compositions and both polarities (Figure 2a,b), the TA devices consistently exhibit higher *g*_m_ values compared to their AC counterparts. For this blend, the *g*_m_ values generally decrease, although not linearly, with the concentration of the respective component. The AC 75:25 P3MEEET:PrC60MA blend is an outlier and shows an increase in n-type *g*_m_ compared to that of pristine PrC60MA despite the reduction of its content in the film. In the P3MEEET:PTEG-1 blend (Figure 2c,d), the p-type *g*_m_ follows the same trend with direct correlation between P3MEEET content and p-type performance and an improvement in all blends upon TA. In contrast, the n-type *g*_m_ does not follow the PTEG-1 content and is notably highest for the 75% PTEG-1 blend. Furthermore, n-type *g*_m_ is generally insensitive to thermal treatment. These results underscore the impact of composition on OECT performance and emphasize the need for a structure–property-performance investigation.

**Figure 2:**
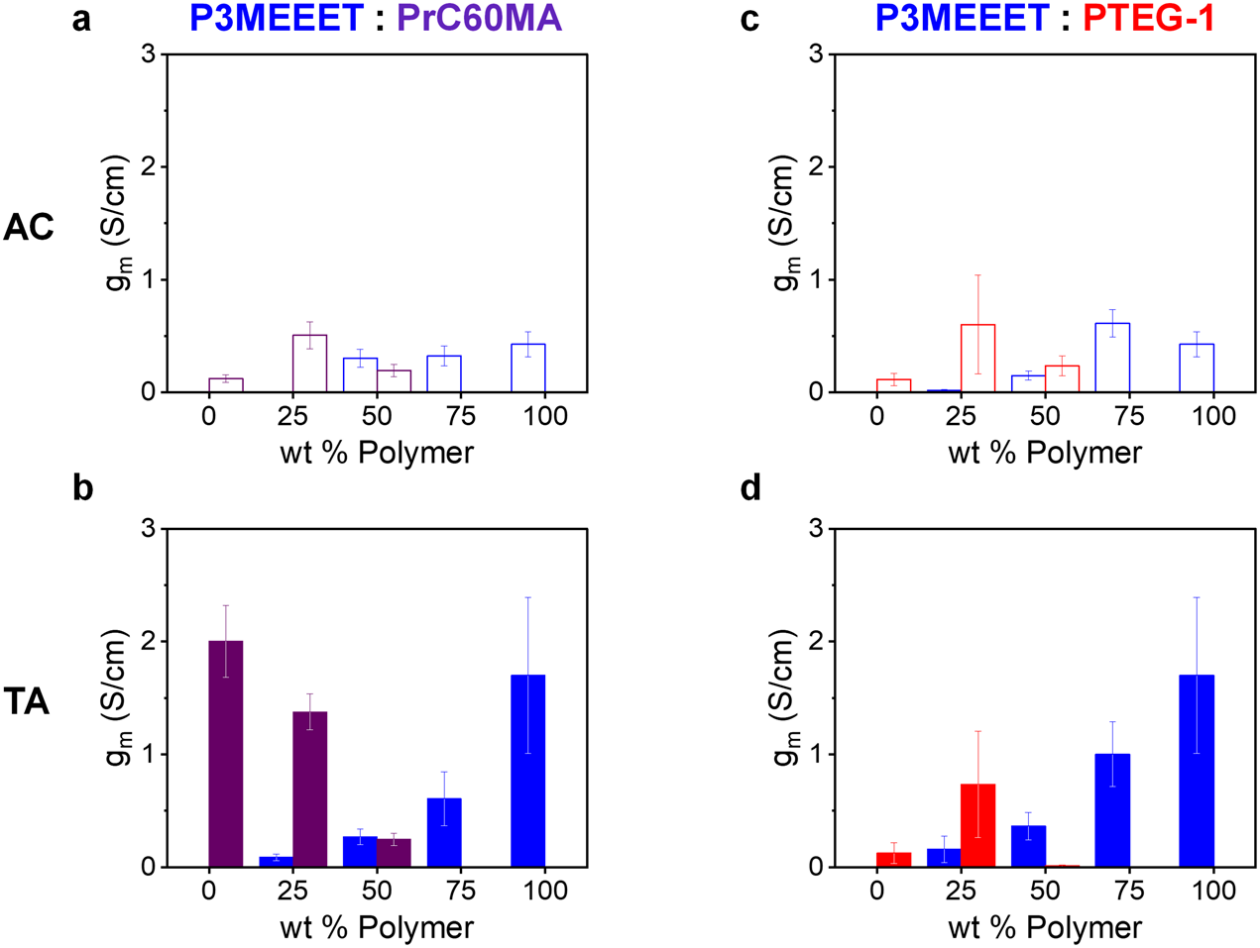
The p- (blue) and n- (purple or red) type transconductance values, *g*_m_, of the P3MEEET:PrC60MA (a, b) and P3MEEET:PTEG-1 (c, d) blends before (top) and after (bottom) thermal treatment.

We start the structure–property performance investigation by analyzing the electrochemical p- and n-doping processes in both blend systems as a function of composition. Cyclic voltammetry (CV) (Figures S6–S8) and spectroelectrochemistry (SEC) (Figures S9 and S10) confirm the unipolar doping of the pristine materials and ambipolar doping across all blend compositions of both systems before and after thermal treatment. The CV shows three distinguishable regions for n-doping, p-doping, and film neutrality at 0 V potential. In the SEC, p-doping induces a clear decay around 500 nm, associated with the P3MEEET neutral π–π∗ transition band, accompanied by the emergence of its polaron peak at ∼750 nm. n-doping leads to broad absorption changes in the 500–850 nm region characteristic of radical anion formation in fullerenes.^59^ Furthermore, the intensities of these spectral and CV changes generally correlate with the concentration of the respective component in the blend, confirming their independent electrochemical response. However, the CV could involve parasitic electrochemical reactions and hence should not be used to evaluate the concentration dependence.

After confirming that each blend component is independently doped, we turned to study the effect of combining the two components on device performance. While transconductance serves as a direct measure of signal amplification, it depends on the physical dimensions of the device and the biasing conditions. The more robust and insightful figure-of-merit to compare materials for the OECTs is the product μC*. This parameter captures the intrinsic mixed transport properties inherent to OMIECs and the potential trade-off between electronic mobility and volumetric capacitance.^60,61^ Therefore, we performed electrochemical impedance spectroscopy (EIS) measurements and extracted the volumetric capacitance (C*) values of AC and TA pristine materials and 50:50 blends for both polarities.

Figure 3 shows that mixing the polymer with either fullerene significantly and similarly reduces p-type C*. A similar reduction is also obtained for the n-type C* in the P3MEEET:PRr60MA blend, with a reduction of the fullerene in the blend. Furthermore, thermal treatments have little to no effect on the C* values of this blend. These results might suggest that for P3MEEET:PrC60MA, the electrochemical properties are simply and strictly concentration-dependent and tunable through the composition. The P3MEEET:PTEG-1 blend shows completely different concentration and thermal treatment effects on C* compared to P3MEEET:PrC60MA (see values in Table S1 and Figure S11 for C* normalized to active material % wt). First, the n-type C* of PTEG-1 and its blend is reduced upon annealing. Furthermore, the value for the 50:50 P3MEEET:PTEG-1 blend is actually higher than that of the pristine PTEG-1. Therefore, we can conclude that although blend composition has a substantial effect on the capacitance of this blend, it is not sufficient to predict or direct device operation, and it is necessary to evaluate the effect of composition and thermal treatments on the charge mobility.

**Figure 3:**
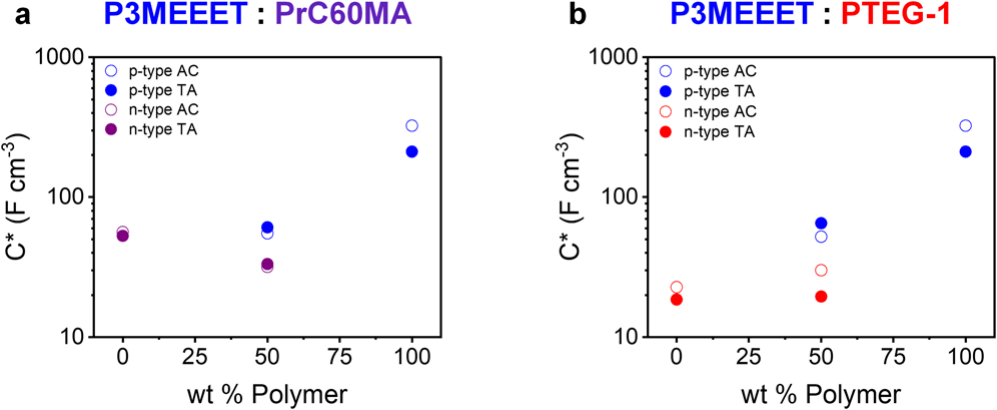
Volumetric capacitance values (C*) of AC and TA pristine materials and 50:50 blends of P3MEEET:PrC60MA (a) and P3MEEET:PTEG-1 (b). Error bars are the same size or smaller than the symbols.

Electronic charge mobility in organic semiconductors strongly depends on molecular ordering, which is often achieved upon thermal treatment, promoting higher charge carrier mobility, ultimately improving device performance.^58,62–64^ Therefore, we utilized grazing-incidence wide-angle X-ray scattering (GIWAXS) to investigate film crystallinity, orientation, and molecular packing. To benchmark the pristine materials and assess the thermal annealing effect, we performed GIWAXS analysis of the AC and TA pristine films. The patterns of AC and TA P3MEEET, in Figure S12, indicate some crystallinity with predominant edge-on orientation and lamellar spacing of 2.08 nm (extracted from out-of-plane line cut, Figure S13) in agreement with earlier studies.^41^ Thermal annealing enhanced ordering, evident by increased peak intensities and a sharper diffraction pattern. The GIWAXS patterns of the pristine fullerenes, Figure S12, show that AC PrC60MA is generally amorphous but crystallizes remarkably upon annealing. The distinct and sharp diffraction spots represent a high degree of tetragonal crystal packing with predominant orientation of the fullerene cages perpendicular to the substrate and fullerene–fullerene spacing of 2.45 nm (Figure S13), in agreement with previous studies.^25,56–58^ In contrast to that of PrC60MA, thermal annealing has little effect on PTEG-1. The GIWAXS patterns of AC and TA PTEG-1 films, Figure S12, exhibit similar diffraction patterns with strong, broadened diffraction spots indicative of an ordered layered structure with fullerene–fullerene spacings of 2.04 and 2.08 nm (Figure S13), respectively, generally aligned along the substrate’s normal direction, as previously reported.^55–57,65^ Therefore, while thermal annealing induces crystallinity and order in P3MEEET and PrC60MA, PTEG-1 is inherently crystalline in the AC film, and thermal annealing has little to no effect on its ordering.

The GIWAXS patterns of the blend films, Figures 4 and 5, show that adding either fullerene to P3MEEET generally does not affect polymer organization, and TA-ordering is maintained. Higher quantities of PrC60MA, 50%, do suppress polymer ordering, while it is still maintained in the PTEG-1 blends (See Figure S15 for peak assignment). The effect of adding polymer on fullerene ordering is completely different for the two fullerenes. For PrC60MA (Figure 4), adding P3MEEET generally disrupts fullerene ordering, which is not significantly affected by thermal annealing. The 25:75 P3MEEET:PrC60MA blend is an outlier to this rule, showing induced PrC60MA ordering upon polymer addition and high order and crystallinity after TA, similar to that observed for the TA pristine PrC60MA film. It is worth mentioning here that the n-type g_m_ of 25:75 P3MEEET:PrC60MA blend was also an outlier (Figure 2), and these results, i.e., induced fullerene order and n-type performance, are possibly correlated.

**Figure 4:**
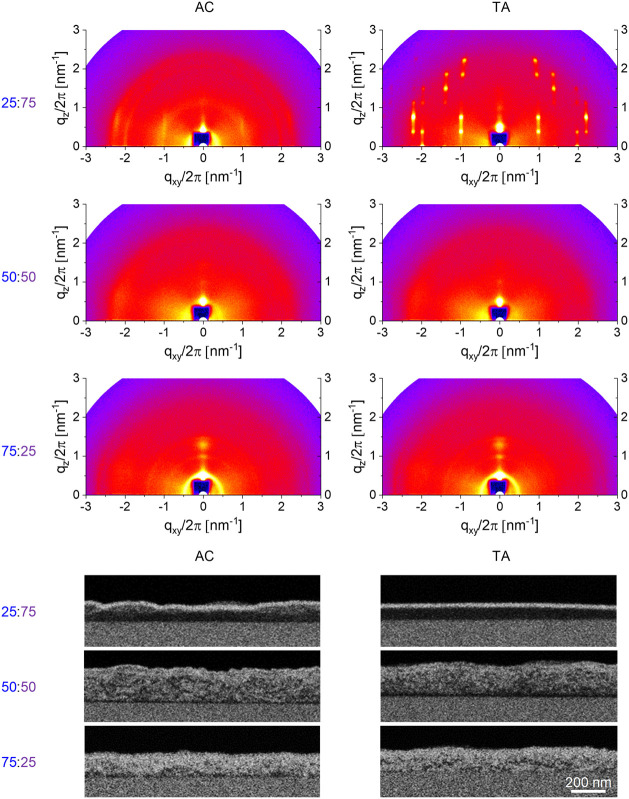
GIWAXS measurement (top) and BSE detector cross-section HRSEM micrographs taken after a VPI process (bottom) of P3MEEET:PrC60MA blend films.

**Figure 5:**
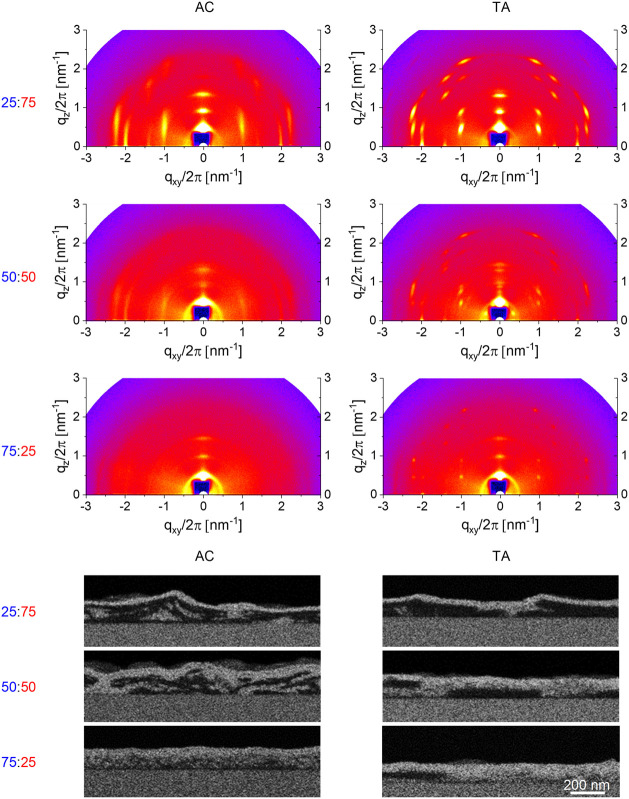
GIWAXS measurement (top) and BSE detector cross-sectional HRSEM micrograph taken after a VPI process of P3MEEET:PTEG-1 blend films.

The effects of TA and composition on fullerene ordering in the P3MEEET:PTEG-1 blends are completely different from those observed for P3MEEET:PrC60MA. Figure 5 shows that adding the polymer to PTEG-1 does not significantly affect fullerene ordering; however, annealing the blends dramatically enhances fullerene crystallization and orientation. For example, the GIWAXS pattern of the AC 25:75 blend is similar to that of pristine AC PTEG-1, but annealing this film transforms the smeared peaks into well-defined diffraction spots, indicating that blending and annealing induces order even higher than that of the pristine PTEG-1. For the 50:50 P3MEEET:PTEG-1 blend, the TA film reveals again a high degree of orientation in the fullerene component. Even for the 75:25 P3MEEET:PTEG-1 blend, TA induces sharp diffraction peaks associated with the PTEG-1 fullerene phase. These results suggest that the presence of the polymer in the blend promotes and facilitates PTEG-1 ordering and crystallization upon annealing.

Although the substantial effect of TA on PTEG-1 ordering is expected to improve n-type charge mobility, the OECT performance of P3MEEET:PTEG-1 blends is generally insensitive to TA (Figure 2). Indeed, Flagg et al. recently showed that charge mobility in OECTs critically depends not only on crystallinity but mainly on connectivity between crystalline domains.^36^ To identify phase distribution and domain size, along with the degree of mixing between the components, we performed Vapor Phase Infiltration (VPI) and cross-section High-Resolution Scanning Electron Microscopy (HRSEM) imaging.^66^ In VPI, the blend films are exposed to gaseous metal oxide precursors that diffuse into the films and in situ convert to an inorganic product, enabling selective “staining” of the polymer (hence, polymer-rich regions appear bright in Back Scattered Electron (BSE) detector HRSEM images) because the precursors diffuse and are retained only in this phase. Fullerene domains are dense and resist precursor diffusion, maintain dark contrast in BSE HRSEM.^35,67,68^ Cross-section HRSEM images of AC and TA pristine P3MEEET, PrC60MA, and PTEG-1 after VPI (Figure S16) verify the infiltration of ZnO precursors into P3MEEET (bright BSE mass contrast) but not into PrC60MA and PTEG-1 (dark contrast), confirming the viability of VPI to map phase distribution in these OECT blends.

The HRSEM images of all P3MEEET:PrC60MA blends show high miscibility between the polymer and fullerene, resulting in a refined BHJ morphology with continuous interpenetrating networks of polymer and fullerene domains (Figure 4). Thermal annealing did not affect the microstructure. In contrast, the HRSEM images of the P3MEEET:PTEG-1 blends (Figure 5) show their strong tendency to phase separate which is enhanced upon thermal annealing. For example, the AC 50:50 P3MEEET:PTEG-1 blend is composed of distinct and developed tens of nanometer long fullerene and polymer domains. Annealing this film prolongs the phase separation to the extent of large, discontinuous, highly ordered fullerene domains. Under these conditions, charge transport across the device channel, while efficient within a fullerene domain, will be limited by domain discontinuity.

## 4 Discussion

Having performed comprehensive electrochemical, electrical, and microstructural analyses, we are now able to analyze the results and elucidate the structure-performance relationships in the blend-based OECTs. The combination of electrochemical insights into doping mechanisms and volumetric capacitance (C*), transconductance (*g*_m_), and the microstructural details revealed by GIWAXS with respect to crystallinity and orientation and by VPI-HRSEM with respect to phase morphology provides a comprehensive picture of the factors governing device behavior in the blend-based OECTs.

The OECT performances of all P3MEEET:PrC60MA blends improve with TA, following the similar behavior obtained for the pristine materials. GIWAXS analysis showed that thermal annealing induces order and crystallinity in P3MEEET and PrC60MA in pristine films and blends, while HRSEM-VPI reveals a highly miscible morphology with finely mixed domains in both AC and TA films (Figure 4). These results suggest that TA-improvement is associated with more effective charge mobility due to enhanced crystallinity of domains for both components, while maintaining phase continuity.

For this blend system, a clear concentration-dependent performance trend emerges (Figure 2) where p-type *g*_m_ generally scales with P3MEEET content, and n-type *g*_m_ generally scales with PrC60MA content. The EIS results show that C* scales with the amount of the respective active material (Figure 3), implying that the volumetric capacitance plays a significant role in this blend system. There is a notable deviation from this concentration-dependent behavior in the AC n-type operation (Figure 2), where the 25:75 blend (the outlier) outperforms that of pristine PrC60MA. GIWAXS revealed that adding the polymer to the fullerene induces fullerene ordering compared to pristine AC PrC60MA (Figure 4), which can promote electron mobility in the blends. This blend-induced ordering was not anticipated during materials selection but was revealed from the results and demonstrates another significant advantage of the blend approach.

In contrast to the P3MEEET:PrC60MA system, the P3MEEET:PTEG-1 blend system shows a less consistent effect of thermal annealing on the performance. While the p-type performance is again improved by annealing (following ordering of the polymer), the n-type performance shows less pronounced or even no response to annealing. This agrees with the behavior of pristine PTEG-1 devices (Figure S1) and GIWAXS patterns (Figure S12) that revealed a negligible impact of thermal annealing on performance and ordering. Therefore, annealing does not induce additional order in the already crystalline PTEG-1 and hence does not enhance n-type mobility and performance. Actually, annealing the 50:50 blend significantly deteriorates its performance. This decay is associated with the extensive phase separation revealed by HRSEM that disrupts the long-range electron percolation pathways from the source to the drain.

OECT performances in P3MEEET:PTEG-1 blends (Figure 2) also deviates from the concentration-dependence obtained for the P3MEEET:PrC60MA system. The p-type *g*_m_ still generally scales with the P3MEEET content in the TA films, but the best n-type performance is achieved for the AC and TA 25:75 P3MEEET:PTEG-1 blends, surpassing the performance of pristine PTEG-1 devices. This improvement is associated with the improved n-type volumetric capacitance (C*) in P3MEEET:PTEG-1 despite the reduction in PTEG-1 content (Figure 3). This enhancement of capacitance is due to the polymer’s assistance in shuttling ions through the film, effectively contributing to fullerene doping via large polymer domains (Figure 5). The improved ionic transport by the polymer is also manifested in the reduced hysteresis in the blend-based n-type transistor behavior (Figure S5). The GIWAXS patterns (Figure 5) indicated that, like the P3MEEET:PrC60MA system, the addition of the polymer induces fullerene ordering and hence charge mobility.

To understand the contrasting behaviors observed between the two blend systems, we turn to the different chemical structures and properties of PTEG-1 and PrC60MA. As mentioned above, PTEG-1 exhibits an inherent strong packing propensity and crystallinity even prior to annealing, with side-chain interdigitation leading to a closely packed crystal structure (Figure S13).^55^ This tightly packed, ordered structure is beneficial for electronic mobility but hinders ionic uptake within pristine PTEG-1 films. Consequently, pristine PTEG-1 devices display comparatively poor OECT performance and minimal response to thermal annealing, as their structure is already highly crystalline and less prone to further ordering that annealing typically promotes. In contrast, the multiple glycol side chains in PrC60MA introduce steric hindrance that stalls self-packing and, in parallel, enhances miscibility with P3MEEET. Thermal annealing induces a relatively spacious packed structure, which, along with the increased number of polar glycol side chains, facilitates ionic uptake and ionic-electronic coupling. Thermal annealing can also introduce some local phase separation and domain ordering, leading to a BHJ morphology with ordered and continuous domains, effectively supporting charge mobility.

It is also of high importance to consider the influence of the p-type polymer’s chemical structure on blend morphology and device performance. Recently, we showed that blends of the non-OMIEC p-type polymer P3HT with PrC60MA in similar concentrations and thermal processing conditions to our present work tend to phase separate, in contrast to P3MEEET:PrC60MA blends.^69^ This distinct difference in miscibility is dictated by the intermolecular interactions; while the hydrophilic P3MEEET promotes hydrophilic–hydrophilic interactions with PrC60MA, the interactions between the hydrophobic P3HT and PrC60MA are hydrophobic–hydrophilic, which drive a lower degree of miscibility and phase separation. Specifically, in P3HT:PrC60MA blends, the polymer migrates to the top of the film, preventing the effective ion infiltration into the bulk. These contrasting morphologies directly influence the percolation pathways for both ionic and electronic charge carriers, consequently leading to distinct operational characteristics for each blend system. Beyond intermolecular interactions, the chemistry of the side chains and the polymer’s molecular weight affect the polymer’s inherent packing propensity and crystallinity.^41,42^ It was shown that lower molecular weight polymers exhibit a greater propensity for crystallization and phase separation.^70^ While maintaining phase continuity within the polymer network is crucial for effective charge transport, polymers typically exhibit a significantly lower percolation threshold compared to fullerenes due to their inherent strand-like characteristics, allowing for effective charge transport even at relatively low concentrations.^71,72^

Comparing the two systems allowed us to establish this qualitative correlation: chemical structure→packing and phase separation tendency→microstructure→OECT performance. We demonstrate this correlation by circling back to the two balanced ambipolar blends: TA 50:50 P3MEEET:PrC60MA and AC 50:50 P3MEEET:PTEG-1. These devices displayed nearly identical balanced ambipolar performance but completely different microstructures:

In the P3MEEET:PrC60MA miscible system, the key factor directing the performance in both polarities is the blend composition. Consequently, the most balanced ambipolar performance in this study is achieved for the TA 50:50 P3MEEET:PrC60MA device due to each component’s inherent unipolar electrochemical performance and the improved performance of both components upon annealing. The BHJ microstructure of this blend is schematically illustrated on the left side of Figure 6. This structure is characterized by intermixed networks of continuous polymer (blue strands) and fullerene (purple spheres) domains. The relatively low degree of crystallinity in this blend, as indicated by the GIWAXS analysis, is visually represented by the ratio of scattered (less ordered) vs packed (more ordered/crystalline) representations of both fullerene “spheres” and polymer “strands.”

**Figure 6:**
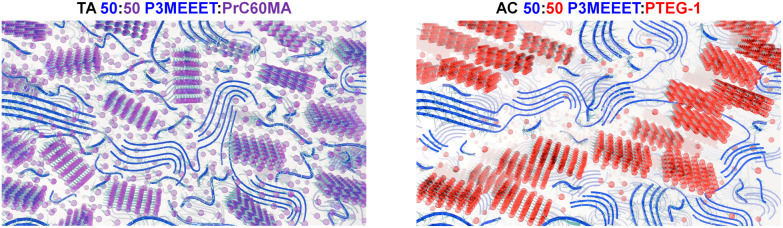
Schematic illustrations of TA 50:50 P3MEEET:PrC60MA (left) and AC 50:50 P3MEEET:PTEG-1 blend (right) microstructures evaluated based on the GIWAXS and VPI-HRSEM results presented in Figures 4 and 5. The blue strands represent the polymer P3MEEET, whereas the purple/red spheres represent the fullerenes PrC60MA and PTEG-1, respectively.

In contrast, the immiscibility of the P3MEEET and PTEG-1 due to the strong tendency of PTEG-1 to self-pack is the dominant factor directing the P3MEEET:PTEG-1 blend microstructure. The microstructure of the AC 50:50 P3MEEET:PTEG-1 film, schematically illustrated in Figure 6 (right-hand side), is characterized by phase-separated large crystalline PTEG-1 domains (red spheres) suspended in a more amorphous matrix composed of large P3MEEET domains (blue strands). In this case, balanced ambipolarity is achieved due to a restrained and optimized degree of phase separation along with the contribution of the polymer to positive ion transport. This comparison clearly demonstrates how the interplay between the composition and microstructure of an OMIEC blend can effectively tune the performance of the OECT across both p-type and n-type polarities to ultimately achieve fully balanced ambipolarity with symmetrical electrical characteristics.

## 5 Summary and Conclusions

This study combined multimodal characterization tools to investigate OMIEC polymer: OMIEC fullerene blends to elucidate the intricate structure–property relationship governing the performance of the OECT and achieve fully balanced ambipolarity. More specifically, we showed that materials with inherent high crystallinity, such as PTEG-1, while offering advantages in charge mobility, may present challenges regarding miscibility and phase separation in blend systems. Conversely, materials with lower self-assembly propensities, like PrC60MA, tend to exhibit better mixing with polymeric components, ultimately leading to concentration-dependent electrical performances for both polarities. Based on these findings, we propose a set of design rules and a framework for achieving balanced ambipolar OECTs using the blend approach. First, informed material selection is crucial, where blend components should exhibit comparable unipolar n- and p-type performances. Ideally, materials should have HOMO/LUMO energy levels aligned with the gate electrode potential and fall within the electrochemical stability window of water. Additionally, leveraging synergistic effects by selecting a polymer with high volumetric capacitance can enhance the capacitive behavior of counter polarity. Phase continuity is key to effective electronic charge transport in both polarities. This can be regulated by selecting blend components with sufficient inherent miscibility, allowing performance tuning through blend composition or by optimizing thermal treatment to avoid excessive phase separation, particularly in systems with crystalline materials. Finally, high charge mobility can be achieved by controlling the crystallinity and order within the blend film. This can be done through thermal annealing and by leveraging blend-induced ordering, where the addition of one component promotes order in the other. We suggest that the principles and framework established here extend beyond the specific materials investigated, offering general guidance for the design of other OMIEC blend systems. The core requirements, i.e., careful material selection based on the energy level position and inherent electronic performance, are universally applicable to OMIEC blends aiming for balanced ambipolar performance. Furthermore, the demonstration of the critical role of microstructure control, particularly phase continuity and crystallinity, represents a general framework suitable for tuning the performance of virtually any OMIEC blend. While the specific microstructures achieved will depend on the unique chemical structures and interactions of the blend components, the principle of controlling and leveraging microstructure to direct performance, as well as the necessity of anticipating and characterizing blend morphology, is universally relevant to OMIEC blends. Beyond the factors explored in this study, we anticipate that parameters such as polymer molecular weight, length, and chemical structure of the side chains in both blend components, and other processing conditions (solvents, annealing time and temperature, etc.), can be used to manipulate microstructure and consequently device performance. Furthermore, the choice of electrolyte is also likely to require optimization for balanced ambipolar performance. In summary, the blend approach opens an exciting and versatile pathway for achieving the next generation of ambipolar polar OECTs, offering unprecedented opportunities to balance and optimize performance through precise microstructure tuning. By applying the outlined design rules, researchers can effectively leverage a wide range of materials systems to develop multifunctional OECTs, which are increasingly in demand for advanced bioelectronic applications.

## Supplementary Material



## Notes

OECT characteristics, cyclic voltammetry, spectroelectrochemistry, volumetric capacitance measurements, GIWAXS patterns and line cuts, and HRSEM micrographs of pristine materials and blends (PDF)
