# Supplementary material for: Balanced Ambipolar OECTs through Tunability of Blend Microstructure
Source: ACS Appl Mater Interfaces. 2025 Jul 18;17(30):43327–38. doi: 10.1021/acsami.5c05400 (PMC12314870; doi:10.1021/acsami.5c05400)
Supplement: Supplementary file 1 [file am5c05400_si_001.pdf]

Supporting information

Balanced Ambipolar OECTs Through Tunability of Blend Microstructure

Noam Moscovich [a], Sasha Simotko [a,b], Efrat Reyn [a], Ido Zerachia [a], Amit Hadar [a] & Gitti L. Frey [a,b*]

a. Department of Materials Science and Engineering, Technion – Israel Institute of Technology, Haifa 32000, Israel

b. The Nancy & Stephen Grand Technion Energy Program, Technion – Israel Institute of Technology, Haifa 32000, Israel

* Email: gitti@technion.ac.il

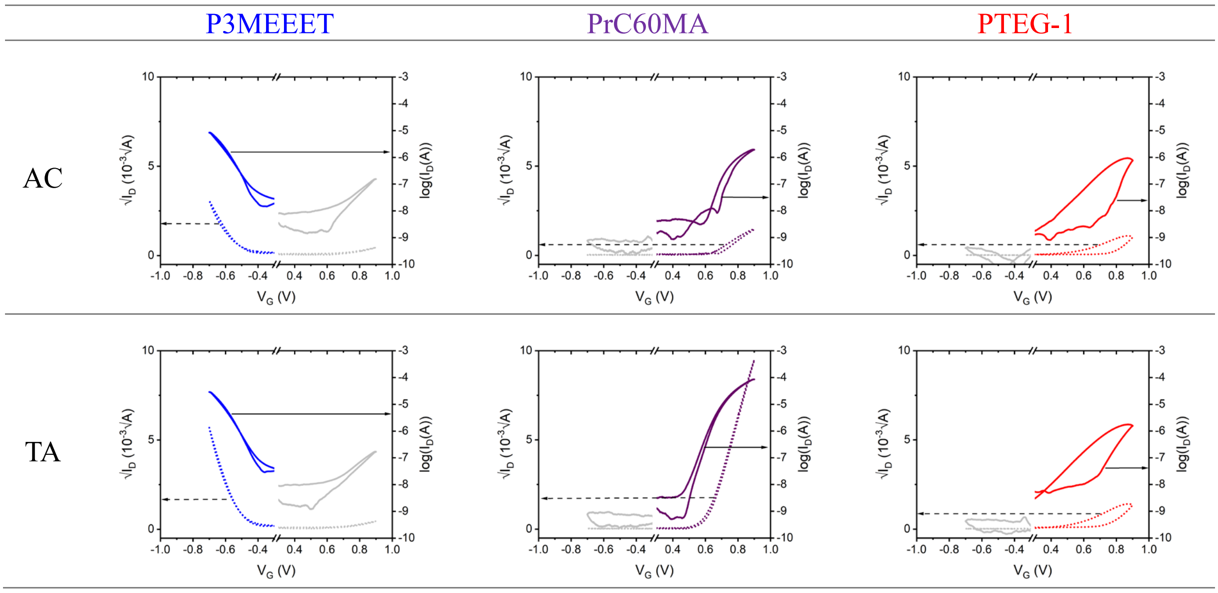

Figure S1: Transfer characteristics of pristine materials OECTs. The colored curves represent device operation in the intended unipolar regime, while grey curves show negligible current modulation in the opposite polarity.

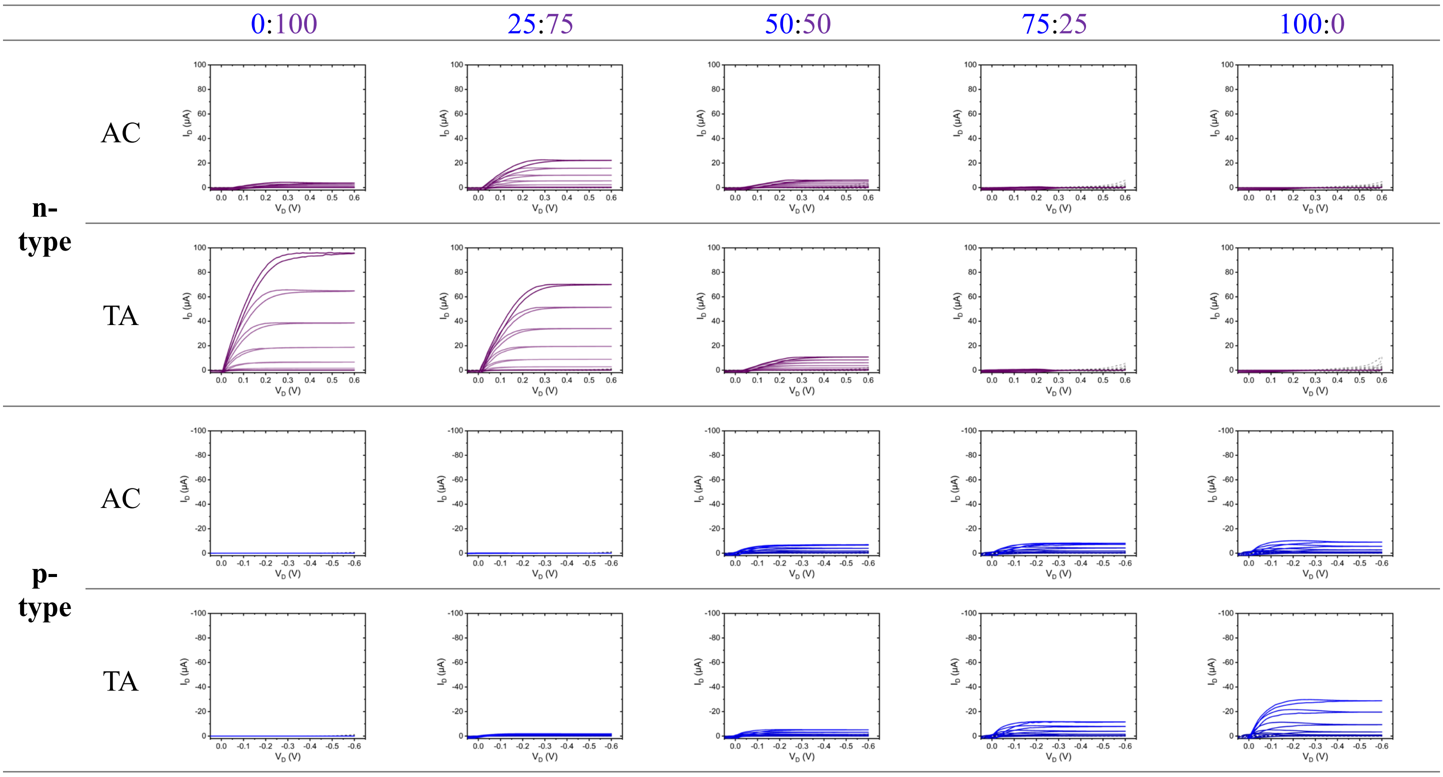

Figure S2: Output characteristics of P3MEEET:PrC60MA blends OECTs.

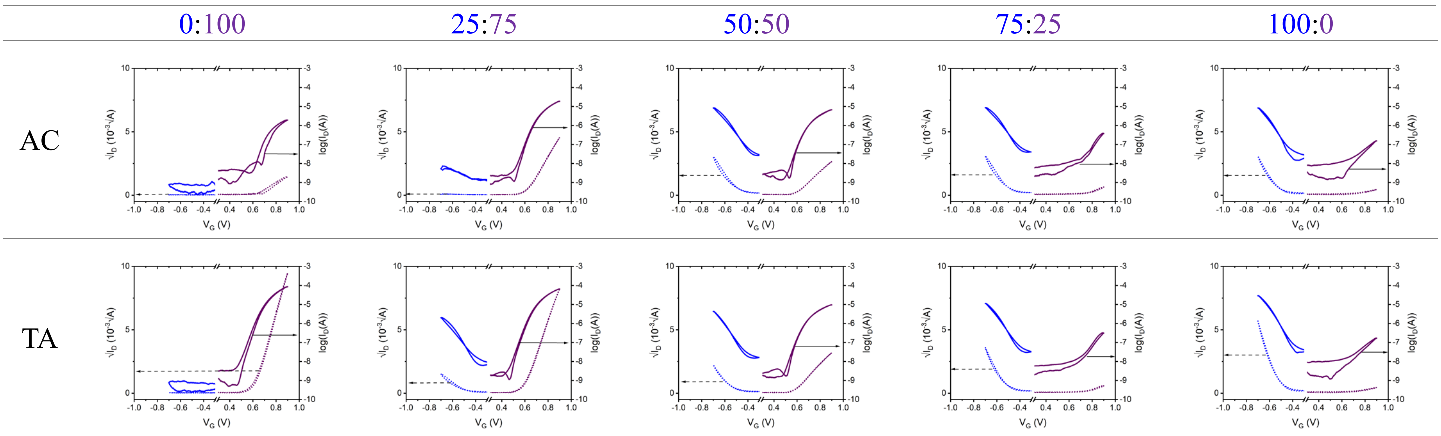

Figure S3: Transfer characteristics of P3MEEET:PrC60MA blends OECTs.

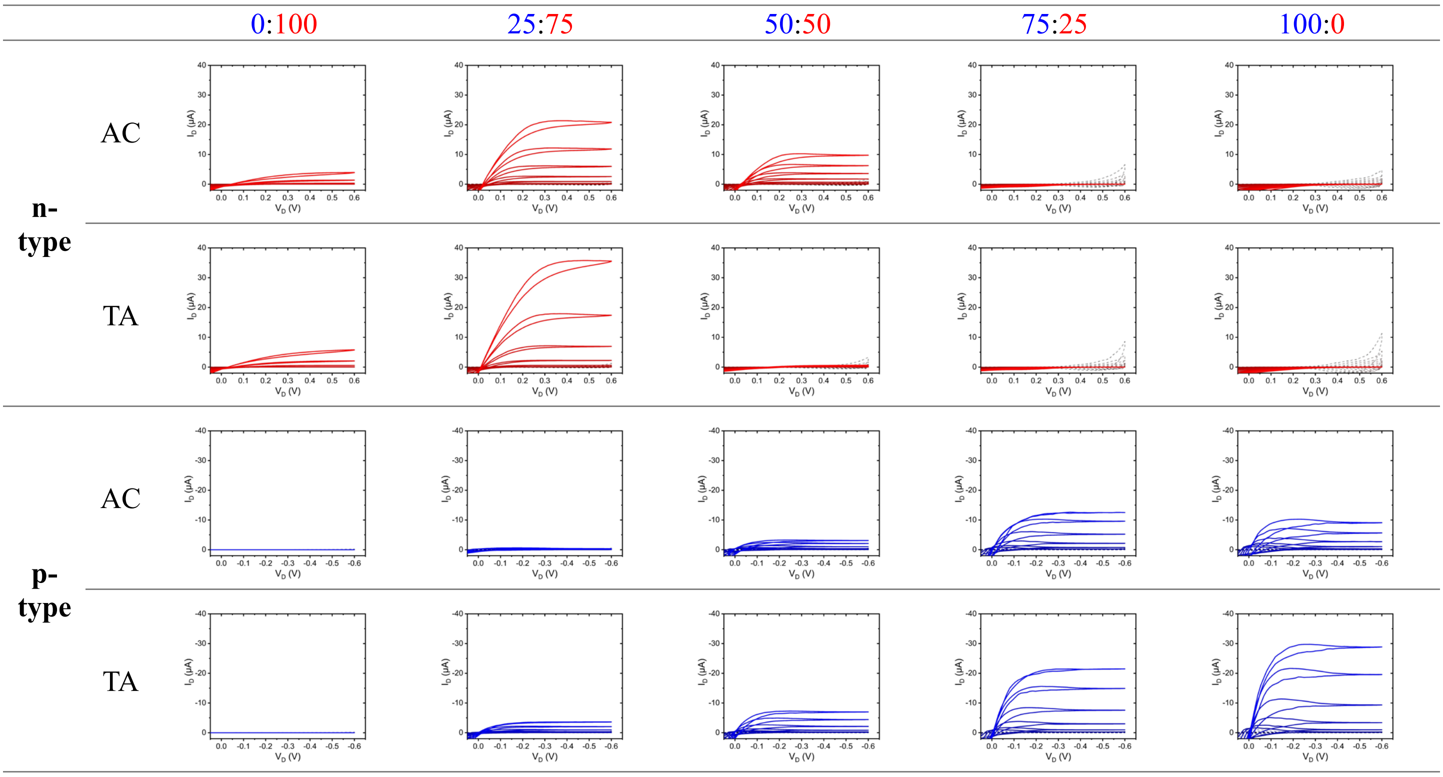

Figure S4: Output characteristics of P3MEEET:PTEG-1 blends OECTs.

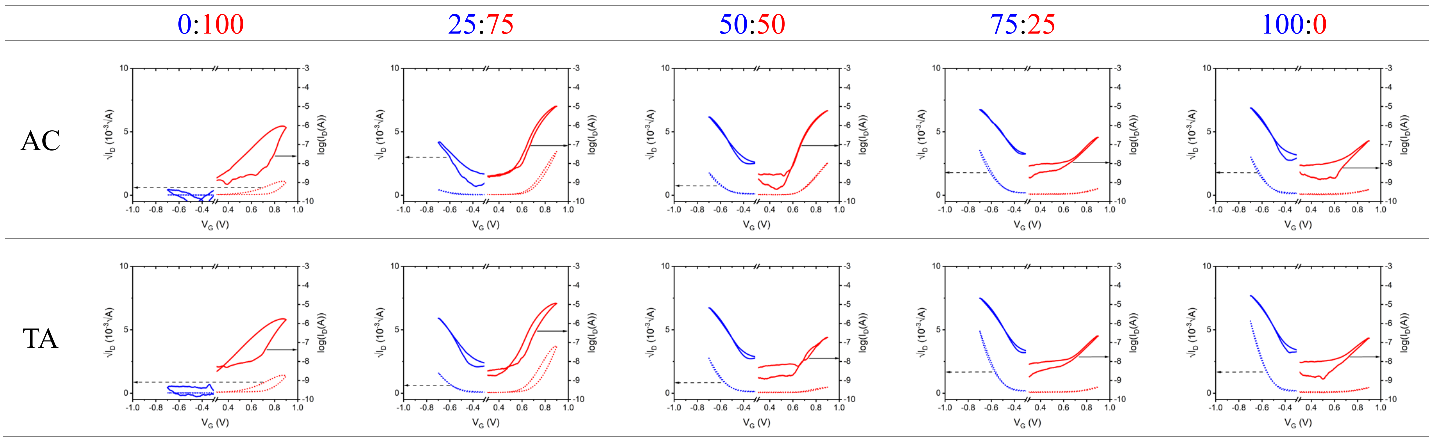

Figure S5: Transfer characteristics of P3MEEET:PTEG-1 blends OECTs.

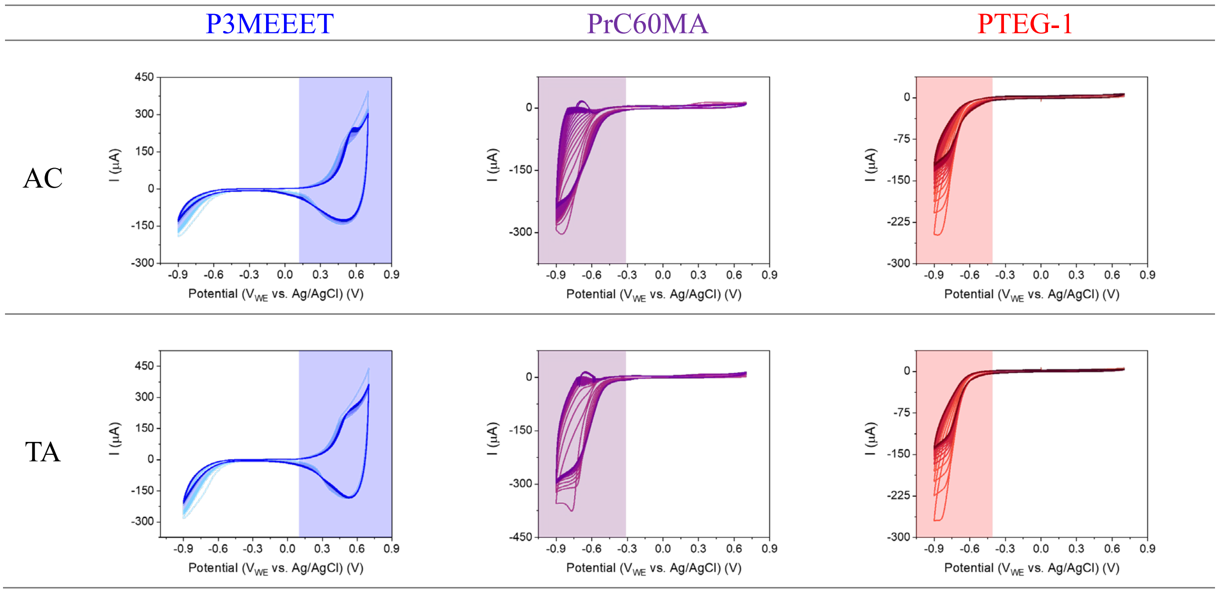

Figure S6: 10 CV cycles of pristine materials AC and TA.

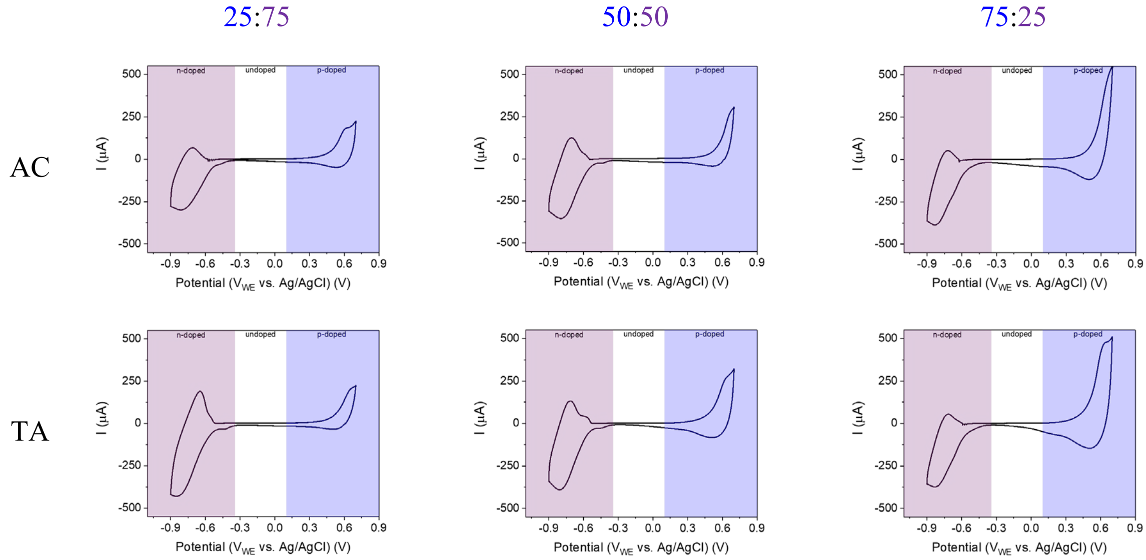

Figure S7: CV of P3MEEET:PrC60MA blends AC and TA. The presented voltammograms correspond to the 10th scan

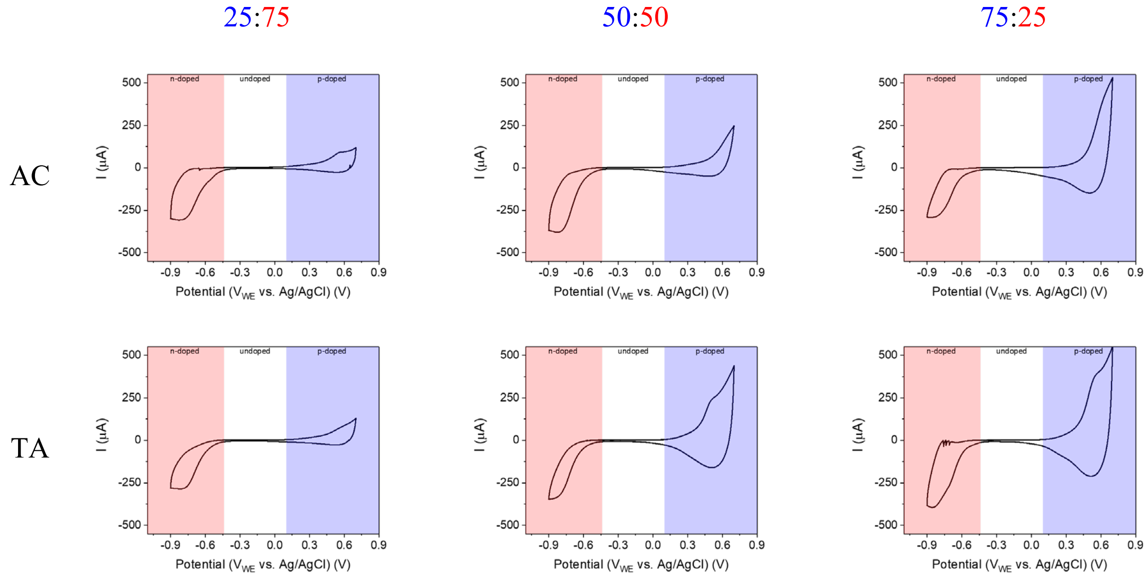

Figure S8: CV of P3MEEET:PTEG-1 blends AC and TA. The presented voltammograms correspond to the 10th scan.

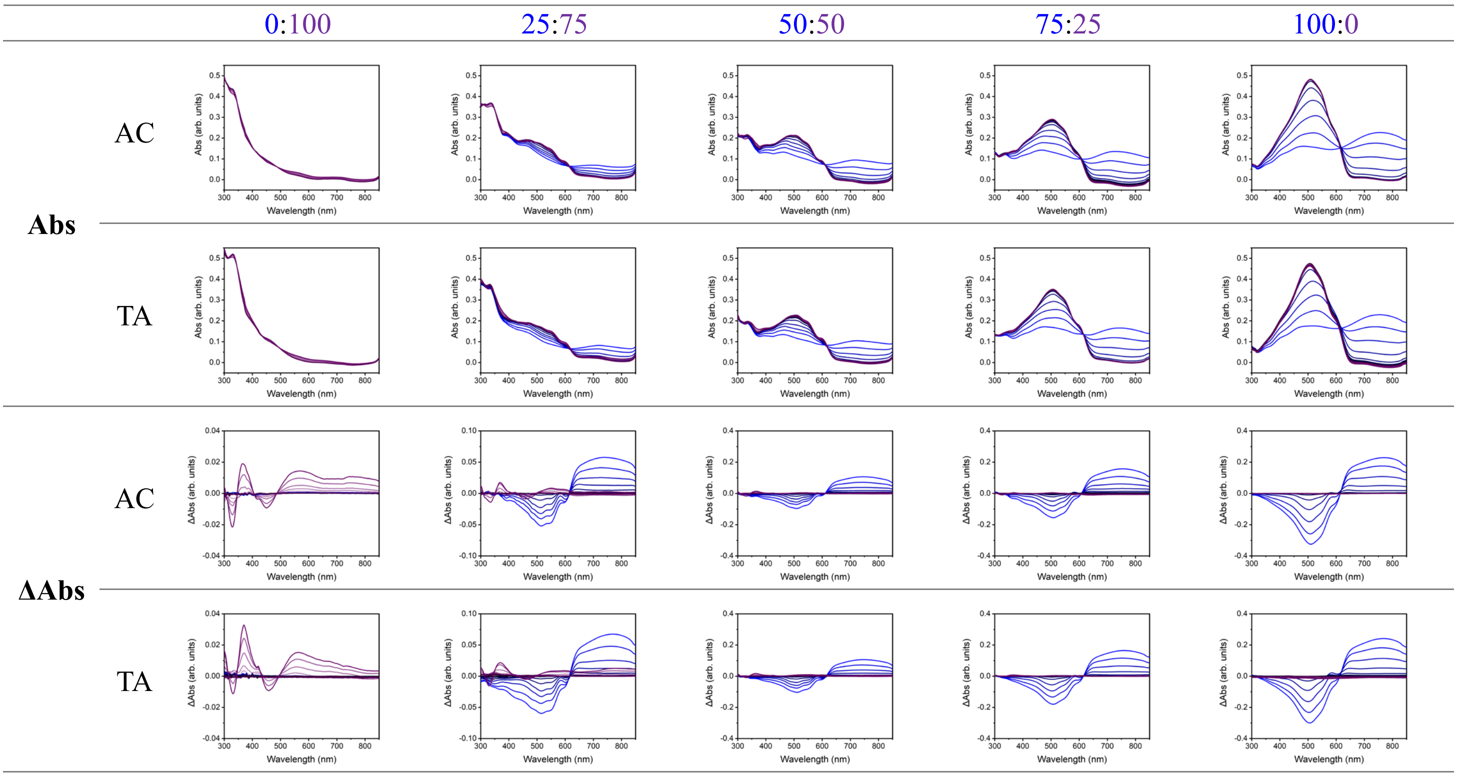

Figure S9: SEC of AC and TA P3MEEET:PrC60MA blends. The displayed absorption changes (ΔAbs) are calculated against the measured spectrum at 0V. Blue curves are representative of positive V_{WE} (p-type doping) and purple curves of negative V_{WE} (n-type doping)

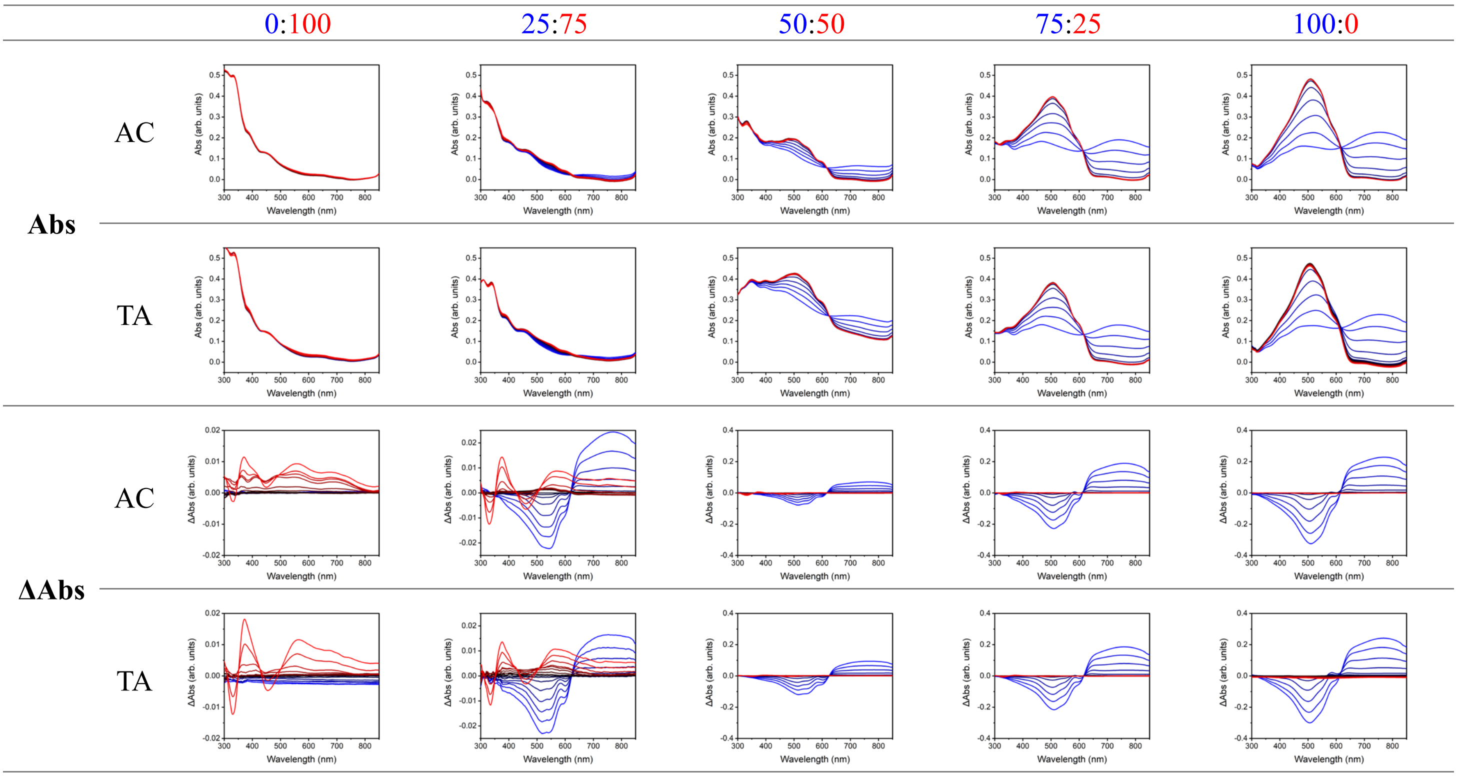

Figure S10: SEC of AC and TA P3MEEET:PTEG-1 blends. The displayed absorption changes (ΔAbs) are calculated against the measured spectrum at 0V. Blue curves are representative of positive VWE (p-type doping) and red curves of negative VWE (n-type doping).

Table S1: Volumetric Capacitance (C) values for pristine materials and 50:50 blends. * denotes no extracted value.*

Film	p-type C* (F/cm^3)		n-type C* (F/cm^3)	
	**AC**	**TA**	**AC**	**TA**
P3MEEET	324.7±25.7	211.4±10.0	*	*
PrC60MA	*	*	56.3±4.2	52.8±1.1
50:50 P3MEEET:PrC60MA	55.1±5.3	60.8±1.9	31.7±3.4	33.2±1.9
PTEG-1	*	*	22.7±0.5	18.5±0.9
50:50 P3MEEET:PTEG-1	52.2±7.0	65.1±3.7	30.1±2.9	19.5±2.6

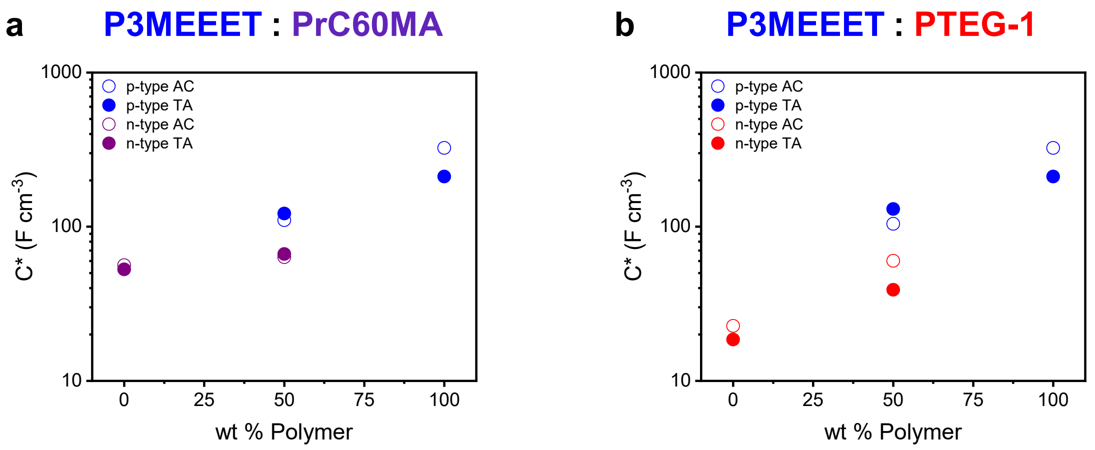

Figure S11: Volumetric capacitance (C) normalized to the wt.% of the respective active material in the blend for P3MEEET:PrC60MA (a), and P3MEEET:PTEG-1(b) blends. n-type C* values are depicted in purple (a) or red (b), while p-type C* values are in blue. Error bars are the same size or smaller than the symbols (The values for the pristine materials are identical to those in Figure 3 in the manuscript).*

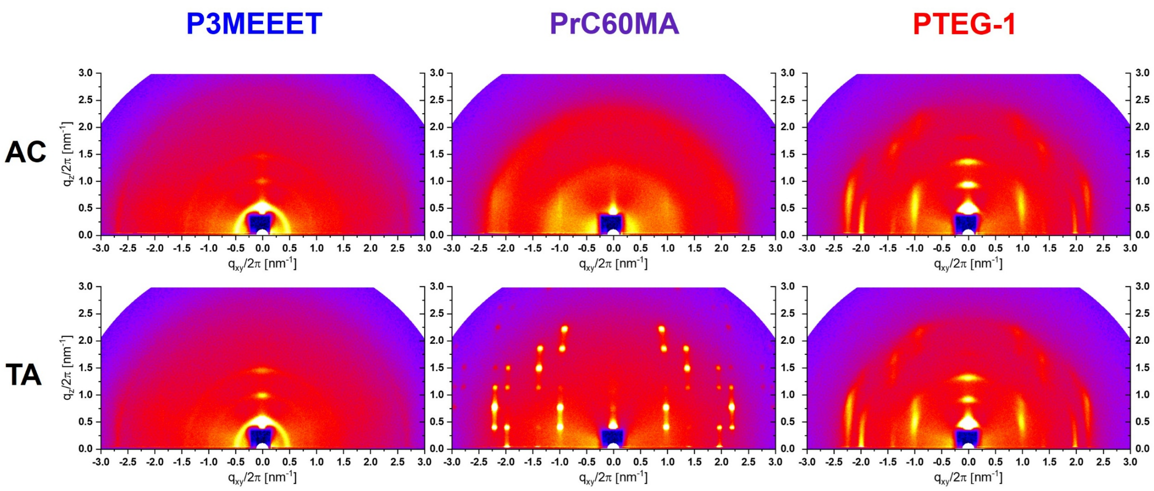

Figure S12: Grazing Incidence Wide-Angle X-ray Scattering (GIWAXS) measurements of pristine materials films.

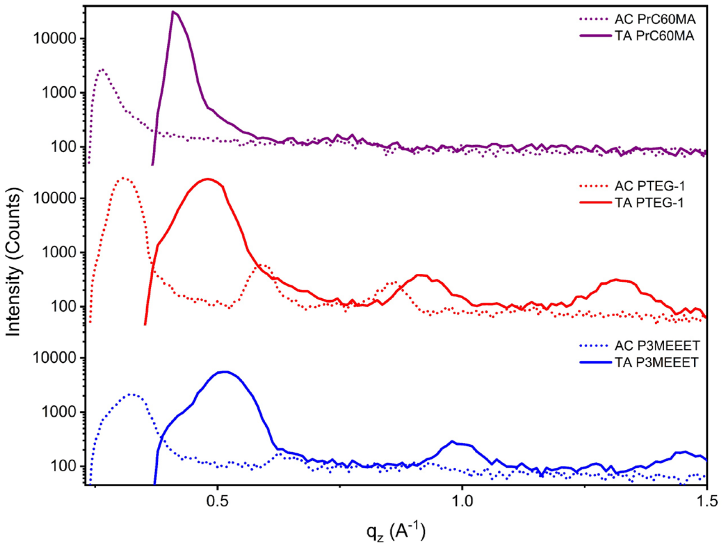

Figure S13: GIWAXS out-of-plane line cuts of pristine materials.

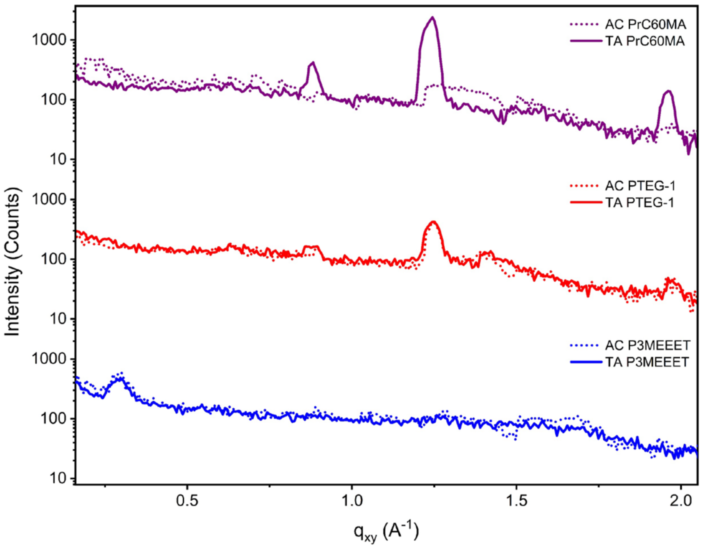

Figure S14: GIWAXS in-plane line cuts of pristine materials.

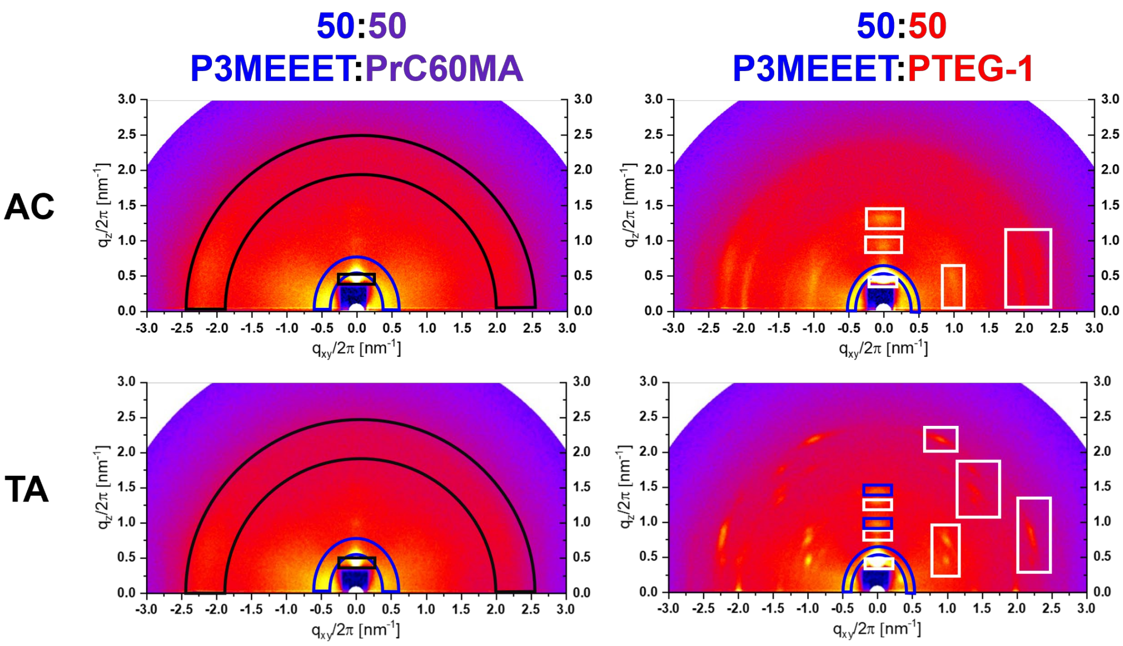

Figure S15: GIWAXS patterns of the AC and TA 50:50 blends. Diffraction spots/rings for P3MEEET are marked in blue, for PrC60MA in black, and for PTEG-1 in white.

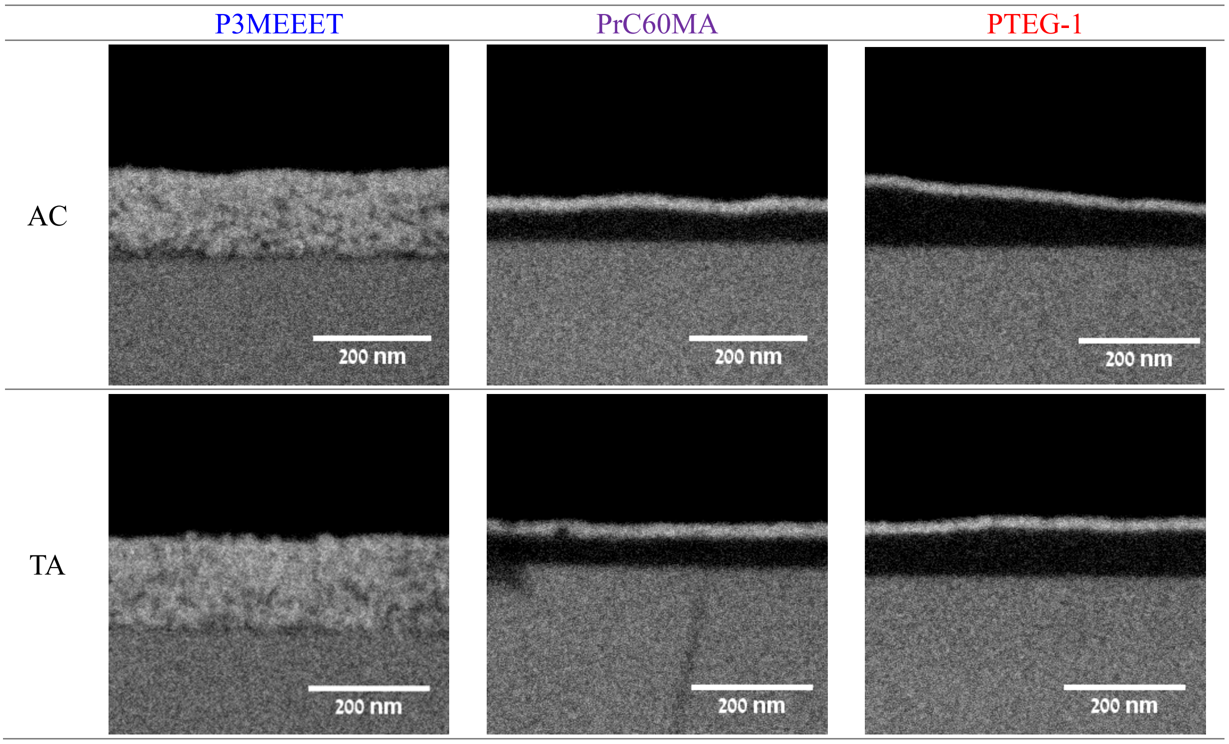

Figure S16: Back-scattered Electrons (BSE) detector cross-section High-Resolution Scanning Electron Microscopy (HRSEM) micrographs of pristine films taken after a Vapor Phase Infiltration (VPI) process.
